# Mesenchymal stem cells as a multimodal treatment for nervous system diseases

**DOI:** 10.1002/sctm.19-0430

**Published:** 2020-06-23

**Authors:** Bogna Badyra, Maciej Sułkowski, Olga Milczarek, Marcin Majka

**Affiliations:** ^1^ Department of Transplantation Jagiellonian University Medical College Cracow Poland; ^2^ Department of Children Neurosurgery Jagiellonian University Medical College Cracow Poland

**Keywords:** immunomodulation, mesenchymal stem cells, nervous system regeneration, neurodegeneration, neuroprotective secretome, stem cells transplantation

## Abstract

Neurological disorders are a massive challenge for modern medicine. Apart from the fact that this group of diseases is the second leading cause of death worldwide, the majority of patients have no access to any possible effective and standardized treatment after being diagnosed, leaving them and their families helpless. This is the reason why such great emphasis is being placed on the development of new, more effective methods to treat neurological patients. Regenerative medicine opens new therapeutic approaches in neurology, including the use of cell‐based therapies. In this review, we focus on summarizing one of the cell sources that can be applied as a multimodal treatment tool to overcome the complex issue of neurodegeneration—mesenchymal stem cells (MSCs). Apart from the highly proven safety of this approach, beneficial effects connected to this type of treatment have been observed. This review presents modes of action of MSCs, explained on the basis of data from vast in vitro and preclinical studies, and we summarize the effects of using these cells in clinical trial settings. Finally, we stress what improvements have already been made to clarify the exact mechanism of MSCs action, and we discuss potential ways to improve the introduction of MSC‐based therapies in clinics. In summary, we propose that more insightful and methodical optimization, by combining careful preparation and administration, can enable use of multimodal MSCs as an effective, tailored cell therapy suited to specific neurological disorders.

## Graphical abstract

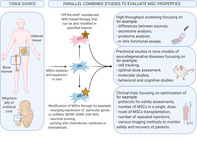

**Significance statement**

This concise review summarizes the results of preclinical and clinical trials in neurological diseases of different etiologies. This review focuses on possible mechanisms of action of mesenchymal stem cells (MSCs) but also discusses approaches to augment their effects. A summary of the properties of MSCs reveals their broad therapeutic potential, which can orchestrate regenerative processes after neural injuries.

## 1 INTRODUCTION

Injury of the nervous system leads to a cascade of events that eventually ends with neuronal loss and acute or chronic dysfunction. Such processes can be caused by neurodegeneration (eg, amyotrophic lateral sclerosis [ALS], Parkinson's disease [PD], and Alzheimer's disease [AD]), autoimmunological reactions (multiple sclerosis [MS]), ischemia (stroke), mechanical injury (traumatic brain injury [TBI], spinal cord injury [SCI]), and other factors (drug‐resistant epilepsy [DRE], cerebral palsy [CP]).^1^, ^2^, ^3^ These diseases impose serious economic and financial burdens on patients, their families, and society as a whole.

Over several years, myths related to the lack of neurogenesis de novo and lymphatic drainage in the nervous system as well as regarding the immune‐privileged state of the nervous system have been debunked.^4^, ^5^ Thanks to recent research, the current view of the central nervous system includes a system that is relatively able to recover. As many diseases and injuries of the nervous system are still untreatable or not efficiently curable by standard medical and pharmaceutical practices, alternatives featuring regenerative medicine might overcome existing barriers.^6^ Transplantation of cells and tissues into the nervous system, which was first performed in the 1980s, aims to promote regeneration through direct replacement of lost cells.^7^ Obtained fetal tissues and implants derived from various sources of neural progenitor cells (NPCs) and neurons still evoke debate around ethical and safety issues.^8^ Apart from the source of new neurons, NPCs have also been shown to have immunomodulatory characteristics.^9^ Nevertheless, discontinuation of the need for treatment with immunosuppressive drugs that comes with allogeneic treatment diminishes or even removes the positive effects of therapies.^10^ Recently, autologous transplantation strategies featuring iPSC technology have appeared.^11^ However, clinical translation of this approach is far from realized because the tumorigenic and long‐term immunogenic potentials of these cells have not been tested.

Strategies for treating diseases and injuries of nervous system appoint a less direct but still beneficial source of cells for transplant to cure such yet incurable diseases—mesenchymal stem cells (MSCs). They can be easily obtained from tissues, expanded ex vivo, and transplanted in an autologous or allogeneic manner.^12^ Due to their immunomodulative properties, MSCs can resolve inflammation triggered by injury or degeneration.^13^ Via their secretome, they can support the survival of neurons and affect the regeneration of tissue loss by influencing local neurogenic niches.^14^

In this review, we introduce unique MSC characteristics valuable for the repair of the nervous system in various diseases based on in vitro and preclinical studies. Taking into consideration the clinical application of MSCs, this review is focused only on the properties of human MSCs from the three most common sources: bone marrow (BM), Wharton's jelly (WJ), and adipose tissue (AT). Clinical studies will be reviewed, focusing on their safety and efficacy. We will also explore discrepancies between clinical studies and suggest potential ways to enhance the effectiveness of MSC therapies.

This narrative review was prepared based on publications found in the PubMed database using the following keywords: MSCs, nervous system, neurodegeneration, and neurological diseases (or each disease specifically, eg. PD, AD, epilepsy, and SCI). For clinical trials, the name of each neurological disease and the term “mesenchymal stem cells” were used as key words, adding “clinical trial” as a filter in the PubMed database. Additionally, clinical trials were filtered from the ClinicalTrials.gov database.

## 2 MSCs AND THEIR PROPERTIES

MSCs, which possess self‐renewal potential and multipotent properties, can be found in neonatal and adult tissues. These adherent, fibroblast‐like cells were first isolated from BM in 1970 by Friedenstein et al.^15^ Over the years, these cells have been called by many names, such as mesenchymal stromal stem cells, multipotent adult progenitor cells, medicinal signaling cells, and mesenchymal progenitor cells (MPCs). Currently, MSCs is the most common terminology, but is sometimes used interchangeably with mesenchymal stromal stem cells to underline their origin from the nonhematopoietic compartment of BM. In addition, MPCs are occasionally presented as a distinct population.^16^

Apart from BM, MSCs have also been identified in AT,^17^ umbilical cord blood,^18^ the umbilical cord lining,^19^ subendothelial layers,^20^ the perivascular zone,^21^ WJ,^22^ dental pulp,^23^ synovial fluid^24^ and the synovial membrane,^25^ amniotic fluid,^26^ fetal liver^27^ and even urine^28^ or endometrium.^29^ Recently, pericytes with MSC‐like characteristics were also found in the brain.^30^

Independent of the tissue source, the isolated cells need to express common characteristics to be defined as MSCs. As no single marker has been specified for these cells yet, analysis of a set of surface antigens needs to be performed. According to the International Society for Cellular Therapy gold standard, MSCs need to be positive for CD73, CD90 and CD105 (all >95%) and negative for CD34, CD45, CD11b/integrin alpha M or CD14, CD79 alpha or CD19, and HLA class II (all <2%).^31^ The status of HLA class II can change upon cell stimulation but the expression of costimulatory molecules, such as CD40, CD80, CD86, CD134, and CD142, cannot be changed.^32^, ^33^ Moreover, the multipotent character of MSCs needs to be proven by their differentiation into adipocytes, chondrocytes, and osteocytes when cultured in vitro.^31^ Some studies suggest that MSCs are also capable of transdifferentiating in vitro to cells outside mesenchymal lineages, such as neural and glial cells, cardiomyocytes, skeletal myocytes, hepatocytes, and endothelial cells; however, these studies have been questioned by recent findings.^34^, ^35^

As a distinct entity from the multipotency understood as differentiation potential per se, the term functional multipotency has been coined.^36^ This characteristic refers to the ability of different types of stem cells to exert pleiotropic influence on injured tissue to support the maintenance of homeostasis, which remains crucial during development but also during tissue repair after injury. Interestingly, studies have shown that sustaining the stemness of MSCs by incorporating specially adjusted scaffolds can highly augment the therapeutic potential of MSCs in treating spinal cord injuries.^37^ Such results should encourage abandonment of the uncertain approach of using transdifferentiated cells.

The most key and characteristic feature of MSCs is their broad secretome, which can influence tissue regeneration. It has been shown that MSCs can produce many immunomodulatory, proangiogenic, tissue remodeling, antiapoptotic, growth, and trophic factors that can support survival of host cells, reconstruction of injured tissue and activation and differentiation of local progenitors.^14^ However, depending on the source of MSCs, they can differ in their properties.^38^, ^39^, ^40^

### 2.1 Differences between sources

Although MSCs from various sources share common characteristics, some differences can be found between them. These variations in MSC populations may reflect particular regional properties of the niches from which they originate.^41^ MSC features are also susceptible to variations between cell culture conditions and isolation protocols. It has been shown that MSCs obtained from the same patient can vary in their properties between isolations. Additionally, discrepancies between different subpopulations of BM‐derived MSCs (BM‐MSCs) have been shown.^42^ In the case of Wharton's jelly‐derived MSCs (WJ‐MSCs), depending on the chosen isolation method (isolated enzymatically by collagenase, trypsin, or hyaluronidase, or by extraction directly from explants), cells can slightly differ in features such as the expression of pluripotency markers and cell proliferation rates.^43^ However, in some reports, it was noted that autologous MSCs differ from those obtained from healthy donors and that such differences can influence the final outcomes of therapies.^44^

Amable et al. published three studies in which the properties of BM‐MSCs, adipose tissue‐derived MSCs (AT‐MSCs) and WJ‐MSCs were analyzed in different culture conditions (medium supplemented with fetal bovine serum or platelet‐rich plasma [PRP]) or during differentiation.^38^, ^39^, ^40^ They confirmed that the higher proliferative potential of WJ‐MSCs compared to cells from other sources was independent of cell culture conditions.^45^, ^46^ AT‐MSCs have a moderate proliferation rate, and BM‐MSCs have the lowest proliferation rate. Authors have shown the influence of the cell culture on MSCs secretome. AT‐MSCs and BM‐MSCs cultured in fetal bovine serum (FBS) produced high amounts of extracellular matrix (ECM) components, which was not observed for WJ‐MSCs. Only AT‐MSCs were able to produce collagen (I, II, and III). However, supplementation of medium with PRP compensated for these differences, although AT‐MSCs were still the only producers of collagen II and IV. Independent of cell culture conditions, BM‐MSCs maintained their highly proangiogenic features. AT‐MSCs cultured in PRP had lower secretion than those cultured in FBS while demonstrating the most pronounced proangiogenic characteristics. Amable et al. also showed that in nonstimulated conditions, WJ‐MSCs produce higher amounts of chemokines able to attract a wide range of inflammatory cells (chemokine ligand 5, monocyte chemotactic protein 1 [MCP‐1], and interferon gamma‐induced protein 10) and interleukin (IL)‐6 than other types of MSCs. The high expression of IL‐6 can be crucial in treating liver fibrosis.^47^ In PRP‐supplemented media, IL‐6 secretion by BM‐MSCs was significantly increased. In other studies, functional tests with phytohemagglutinin‐activated T lymphocytes or peripheral blood mononuclear cells have shown that BM‐MSCs are the most immunosuppressive cells. This feature has been preserved independently of cell culture conditions.^45^, ^48^, ^49^ Interestingly, Amable et al. also evaluated how the levels of secreted chemokines, cytokines, ECM proteins, proangiogenic factors, and growth factors changed in differentiated cells. Those experiments have shown that some characteristics are preserved, for example, high expression of vascular endothelial growth factor (VEGF) by BM‐MSCs and very low expression of ECM proteins by WJ‐MSCs, whereas some of the characteristics dramatically changed, for example, expression of interleukins or collagen II by differentiated AT‐MSCs.^40^ These results show that the differentiation of cells can influence some properties of MSCs. These findings also underlie the importance of cell culture conditions on the final properties of cells that can be later administered to patients.

## 3 MSCs IN NEURODEGENERATION AND BRAIN INJURY—PRECLINICAL TRIALS

The previously mentioned features of MSCs make them a perfect tool in cellular therapies for pathological processes in the nervous system driven by excessive inflammation and neurodegeneration. MSCs secrete a variety of factors, including neurotrophic factors.^50^ MSCs from different sources can also differentiate into neuronal lineages by forming primary neurospheres; however, only WJ‐MSCs and BM‐MSCs could form secondary neurospheres.^51^ Moreover, differentiated WJ‐MSCs secreted more neurotrophic factors than BM‐MSCs and AT‐MSCs.^51^

### 3.1 Routes of MSC transplantation

Preclinical studies have established ways of MSC implementation via intravenous, intra‐arterial, intrathecal, intranasal, intraperitoneal, intraspinal, intracerebroventricular, intracerebral, or direct administration to particular structures. The route of administration is important because it determines the number of successfully grafted cells in the injured site, which can be correlated with therapeutic outcome.^52^, ^53^ Additionally, taking into consideration that neurological disorders may not be localized, indirect administration, for example, intrathecal injection, may be of great importance.

To understand the pros and cons of each route of administration, an invaluable tool is cell tracking. Various methods have been developed for intravital imaging.^54^ Studies have shown that although the most feasible method of MSC transplantation is through intravenous injection, in such conditions, most of the cells become trapped in the lungs.^55^ Nevertheless, such entrapped MSCs can release microvesicles and immunomodulative factors and affect the overall state of the patient by modulating peripheral immune cells.^56^ Moreover, cell tracking can visualize MSC migratory potential.

### 3.2 Active migration of MSCs toward injury

Once administered indirectly, MSCs need to actively migrate to the injury region. Active migration of MSCs is possible due to the expression of receptors and cell adhesion molecules. Pivotal roles are played by receptors, integrins, selectins and proteolytic enzymes.^54^ One of the pathways crucial for MSC migration is the METR/HIF‐1/CXCR4 pathway.^57^ It was shown that preconditioning MSCs with stroke patients' sera enhanced the METR/HIF‐1/CXCR4 pathway and increased the migratory potential of MSCs, which translated into improved recovery in a transient middle cerebral artery occlusion (tMCAO) stroke model in rats.^57^ Important chemoattractants that can enhance MSCs to regions of injury in the brain are MCP‐1 and stromal cell‐derived factor‐1 (SDF‐1). In a study by Lee et al., it was shown in the MCAO rat model of stroke that MCP‐1 and SDF‐1 have both region‐ and time‐dependent differential expression, which directs intravenously injected MSCs to migrate either to the cortex 1 day after injury or to the striatum in later days.^58^ That MSC migration dependent on CXCR4 receptor expression was also shown in elegant in vitro studies with microfluidics systems.^59^

### 3.3 MSCs act through immunomodulation

MSCs are mostly recognized as immunomodulatory cells that can balance inflammation in the tissue environment by upregulating anti‐inflammatory signaling and decreasing pro‐inflammatory signaling and thus regulate immunological cells such as lymphocytes, macrophages or microglia and astrocytes.^60^, ^61^ MSCs can influence inflammation by secreting soluble factors or direct cell‐cell contact. MSCs constitutively or upon stimulation secrete indoleamine 2,3‐dioxygenase, transforming growth factor beta, hepatocyte growth factor, IL‐6 and IL‐10, prostaglandin E2, heme oxygenase 1 and soluble HLA‐G5.^13^

Inflammation is an integral part of pathological processes that emerge in the nervous system. Depending on the mechanism of injury or neurodegeneration, the innate or adaptive system plays a more important role. Due to their migratory potential, MSCs can migrate to the site of injury and, due to their immunomodulatory properties, decrease inflammation. Excessive inflammation in the brain is the most devastating force causing degeneration of the central nervous system.^62^, ^63^ Acute injuries due to ischemia (hypoxia‐ischemia encephalopathy [HIE] and stroke), mechanical‐driven trauma (such as TBI) or progressive neurodegeneration lead to moderate activation of microglia followed by activation of astrocytes, the main sources of inflammatory cytokines.^64^ If this state persists, damage to the blood‐brain barrier (BBB) occurs, and intensified inflammation appears due to the migration of peripheral immune cells (lymphocytes and monocytes).^65^ Such processes suppress neurogenesis and endogenous repair.^66^ Thus, MSCs could be a perfect tool in brain injuries, as they can modulate the inflammatory state. It has been shown that MSCs can attenuate microgliosis and astrogliosis in rats with induced HIE, SCI, or epilepsy.^67^, ^68^, ^69^ MSCs can also suppress the proliferation and differentiation of B lymphocytes.^70^ Moreover, transplanted MSCs can switch activated M1‐phenotype microglia to the regenerative M2 phenotype.^71^, ^72^, ^73^ Additionally, in an AD model of APP/PS1 double transgenic mice, transplantation of MSCs led to reduced β‐amyloid (Aβ) peptide deposition by microglia but without secretion of proinflammatory factors.^73^, ^74^, ^75^ MSCs have also been shown to improve BBB integrity in a rat TBI model.^76^ This effect was mainly mediated by the activity of metalloproteinase inhibitor 3 (TIMP‐3) released by MSCs.

### 3.4 MSCs support regeneration through their neurotrophic activity

Decreasing the inflammation status may contribute to the onset of direct repair of neuronal circuitry and activation of endogenous neurogenesis by MSCs. It has been shown that timing of extinguished inflammation and activation of local progenitors overlap in MSC‐treated mice with induced HIE.^71^ MSCs and MSC conditioned media (MSC CM) alone can activate local progenitors in healthy adult rat brains and their differentiation into immature neurons in the subventricular zone (SVZ).^77^ However, injection of MSC CM into the brain alone cannot protect the brain against inflammation and has a short‐lasting effect. Moreover, repeated transplantations of MSCs in a D‐galactose‐induced mouse model of cognitive decline have shown functional improvement measured in cognitive tests by enhancing synaptic plasticity and endogenous neurogenesis.^78^ This effect was shown to be mediated by activation of the mitogen‐activated protein kinase‐ERK‐CREB signaling pathway in the aged hippocampus. Transplantation of MSCs also rescued long‐term potentiation impairment in aging mice through impacts on electrophysiology.

The neuroregeneration ability of MSCs is also based on the secretion of a wide range of paracrine substances by host cells and MSCs. Several growth factors are secreted by MSCs: brain‐derived neurotrophic factor (BDNF), nerve growth factor, insulin‐like growth factor 1, glial cell‐line‐derived neurotrophic factor (GDNF) and VEGF.^79^, ^80^, ^81^ The neuroprotective function of transplanted MSCs is based on a reduction in neuronal sensitivity to glutamate receptor ligands and altered gene expression, suggesting a link between the therapeutic effects of MSCs and the activation of cell plasticity in damaged nervous structures.^82^ Experimental models proved that the MSC secretome promotes axonal growth and neuroprotection and minimizes cavity formation in SCI.^83^, ^84^ Neurotrophic and neurotropic effects of MSCs were also clearly presented in some elegant ex vivo studies employing adult rat dorsal root ganglia organotypic cultures.^37^, ^85^ Interestingly, this type of culture can be used to decipher other effects of MSCs on injured tissue, for example, immunomodulation.^37^ Lu et al. investigated the nature of chronic scars and their ability to block axon growth. Chronically injured spinal cord axons can regenerate through the gliotic scar in the presence of local growth‐stimulating factors.^86^ MSCs may provide a source of growth factors to enhance axonal elongation across spinal cord lesions and minimize cavity formation in SCI.^68^, ^87^ Interestingly, growth factor secretion, neurogenesis, and survival of stem cells are improved when MSCs are harvested under hypoxic conditions.^88^

### 3.5 MSC modifications and neuronal priming

Despite their innate, multipotent character, MSCs can be even more perfectly tailored to the exact type of injury. This advancement can be achieved by appropriate MSC preparation for the specific environment before transplantation. Up to date, various ways of priming of MSCs with cytokines, hypoxia, pharmacological agents, biomaterials, and other molecules have been established (comprehensively reviewed in Reference 89).

In some neurological disorders, such as PD, ALS, and stroke, gene therapies are proposed as a method of treatment.^90^ One of the challenges of such approaches is the delivery of genes of interest, especially in nonfocal neurodegenerations. MSCs can thus serve as carriers for genes whose expression is needed in specific neurological disorders: GDNF (PD and ALS),^91^, ^92^ VEGF (PD),^93^ GDNF and VEGF (ALS),^94^ BDNF (Huntington's disease [HD], SCI, and stroke),^95^, ^96^, ^97^ conserved dopamine neurotrophic factor,^98^ and PlGF (stroke).^99^ It has been shown that modified cells have a more pronounced therapeutic effect than unmodified MSCs. This result stems from the fact that despite the desired alterations, modified MSCs maintain the rest of their characteristics, such as a capacity for immunomodulation. However, more studies are needed to determine whether transient delivery of such growth factors is sufficient or repeated transplantation is needed depending on the treated disease. In contrast, MSCs can also be used to decrease undesirable genes to promote repair. One study has shown that modification of MSCs with lentiviral RNAi downregulating adenosine kinase, the major adenosine‐removing enzyme, may be beneficial for treating epilepsy. Indeed, transplantation of such modified MSCs resulted in a decrease in seizures, and this effect was strictly connected to elevated levels of adenosine.^100^ Moreover, modified MSCs transplanted directly into the injury site demonstrated a better ability to promote neuron survival and decrease damage than unmodified MSCs.^101^

Additionally, taking into consideration the impact of miRNAs in the regeneration of tissue, MSCs can serve as carriers of different miRNAs to the nervous system.^102^, ^103^ However, in some cases, a decrease in miRNAs can be beneficial. For example, in the case of SCI, suppression of miR‐383 enhances the therapeutic potential of MSCs in SCI.^104^

There have also been studies with an established cell line, SB623 hBM‐MSCs (SanBio Inc.), which overexpresses the NOTCH 1 intracellular domain. These cells transplanted in preclinical trials have ameliorated damage in models of PD or after TBI,^105^, ^106^ probably due to enhanced neuropoietic and proangiogenic activity.^107^, ^108^ Moreover, in a TBI model, transplanted SB623 cells formed “biobridges” between the neurogenic niche and the site of injury.^106^ This effect has been correlated with metalloproteinase‐9 (MMP‐9) activity. Functional formation of these biobridges can play an important role not only in TBI but also in ischemic injuries in the brain.^109^ These results can partially explain the regenerative potential of SB623 cells in clinical trials with stroke patients.^110^ Currently, a clinical trial is underway in which SB623 cells are being applied to treat patients with TBI (STEMTRA, NCT02416492).

MSCs have been shown to differentiate into many types of cells from the nervous system, including dopamine neurons,^111^ acetylcholine‐secreting motor neuron‐like cells,^112^ cholinergic‐like neurons,^113^ GABAergic neurons,^114^ and oligodendrocytes.^115^ However, the functionality of these cells, manifested by, for example, the ability of the cells to be engrafted into recipient models and to form connections with existing circuits, remains controversial. Notably, the conditions required to force MSCs to differentiate into neuronal cells necessitate the presence of factors, such as 5‐aza‐deoxycytidine, that are not naturally found in living organisms. On the other hand, priming the MSCs to a neural phenotype may pronounce the therapeutic effect of MSCs alone. The differentiation potential of MSCs can be exploited during their preparation before transplantation. It was shown in a PD model that neuro‐primed MSCs have an enhanced restorative effect.^116^, ^117^ Such an effect was also observed in MSCs that were primed by overexpression of Lmx1α and neurturin, which are important factors for differentiation and survival of dopaminergic neurons.^118^ Another method of genetic priming features overexpression of neurogenin 1 (Ngn‐1) in MSCs. These cells have pronounced therapeutic effects in a mouse model of ALS and brain ischemia in comparison to unmodified MSCs.^119^, ^120^ Although MSCs overexpressing Ngn‐1 exhibited neuron‐like characteristics in these studies, all transplanted cells vanished within 8 weeks. Additionally, the authors have not yet evaluated the electrophysiological recordings of MSCs after transplantation, which will support their claims. Other preclinical studies have also demonstrated that MSCs are more likely to differentiate into neuronal‐like cells when in the presence of other neuronal cells.^121^

### 3.6 MSCs cotransplantation with other cells

MSCs can also be immunosuppressive in xenotransplantation models. Intrastriatal cotransplantation of syngeneic MSCs with porcine neuroblasts into 6‐OHDA unilaterally lesioned rats resulted in successful neural stem cells grafting in four out of six rats.^122^ Beyond changes at the cellular level, motor recovery was also observed due to transplantation. This result suggests that MSCs can be used in xenotransplantation instead of immunosuppressants, for example, cyclosporin A, which cause side effects and are not able to protect long‐lasting grafts. On the other hand, in rat models of PD, it has been shown that human MSCs can evoke inflammation after transplantation. This may be due to differences between the characteristics of syngeneic and xenogeneic transplanted MSCs. These data suggest that the immunomodulatory effect can be inefficient in xenotransplanted MSCs or that other mechanisms were triggered in those studies.

**FIGURE 1:**
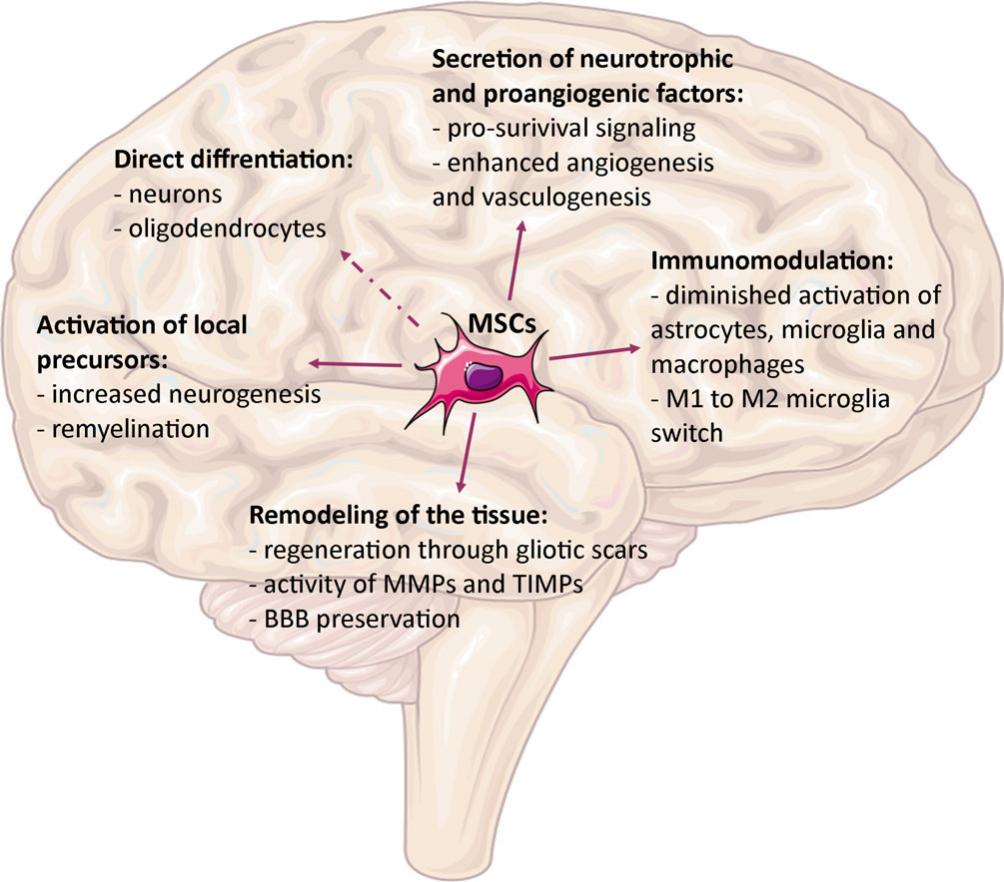
Therapeutic mechanisms of mesenchymal stem cells (MSCs) in the nervous system. Source: Servier Medical Art, modified. BBB, blood‐brain barrier

The aforementioned interactions of MSCs with diseased nervous tissue, including modulation of inflammation and neurogenesis by MSCs, seem to be the key to modifying the environment to pursue regeneration (Figure 1). The multimodal activity of transplanted MSCs in models of neurodegenerative diseases has led not only to a pathophysiological view of the course of disease but also, in some cases, to a therapeutic effect, such as better cognitive outcomes in models of AD, better motor activity in models of PD and ALS or a decrease in the amount and severity of recurrent seizures in epilepsy.^119^ Understanding which conditions are crucial to boost efficiency by optimizing the route, time and number of administrations of MSC transplantations in preclinical models will improve our knowledge and enhance translation into clinical trials.

## 4 CLINICAL TRIALS

Selected clinical trials using MSCs to treat neurodegeneration and brain injury are presented in Table 1. In the numerous presented studies, expanded ex vivo autologous MSCs were transplanted. However, in a clinical trial for CP, neural‐primed autologous MSCs were used, and allogeneic administration of the previously mentioned SB623 cells in stroke patients has been described.

**TABLE 1:** Selected clinical trials in neurodegeneration and brain injury involving mesenchymal stem cells (MSCs)

Disorder	Trial number	Phase	Type of trial	Cells applied	Amount of cells	Time from onset	Delivery method	Results	Reference
TBI	—	—	Nonrandomized, uncontrolled	Auto BM‐MSCs	1° 1 × 10^7^‐1 × 10^9^ 2° 1 × 10^8^‐1 × 10^10^	Undisclosed	1° at injury site (intracerebral) 2° intravenous (after 4‐12 days)	Safety (CTCAE v3.0); improved scores of BI	123
TBI	—	—	Nonrandomized, uncontrolled	Auto BM‐MSCs	1 × 10^6^	Undisclosed	Intrathecal	Safety; improvements in motor functions (27/73), improvements in consciousness (PVS; 11/24); better results for younger patients and for therapies earlier after injury	145
Stroke	NCT01287936	I/IIa	Nonrandomized, uncontrolled	Allo SB623 (modified BM‐MSCs)	2.5‐10 × 10^6^	7–36 mo	At injury site (intracerebral)	Treatment‐emergent adverse events without sequelae (18/18); improvements in the mean scale scores of ESS, NIHSS, F‐M total score, and F‐M motor function total score	110
Stroke	—	I/II	Randomized, controlled	Auto BM‐MSCs	1‐2° 1 × 10^8^	32‐61 days	1‐2° intravenous (1°‐4 to 5; 2°‐7 to 9 wk after symptoms onset)	Safety; improved scores of BI	133
Stroke	—	—	Randomized, controlled, observer‐blinded	Auto BM‐MSCs	1‐2° 1‐2 × 10^7^	~5 wk	Intravenous (2° 2 wk later)	Safety; increased number of patients with mRS scores 0‐3	134
Stroke	—	I	Nonrandomized, uncontrolled	Auto BM‐MSCs	0.6‐1.6 × 10^8^	36‐133 days	Intravenous	Safety; improvement in NIHSS scores	135
Stroke	—	—	Nonrandomized, controlled	Auto BM‐MSCs	5–6 × 10^7^	7‐12 mo	Intravenous	Safety	127
CP	ChiCTR‐TRC‐12002056	—	Nonrandomized, controlled, observer‐blinded	Auto neural‐primed BM‐MSCs	1‐2° 1‐2 × 10^7^	1–32 yr	Intrathecal (2° 3 wk later)	Safety; motor recovery (better in group with GMFCS levels IV and V)	136
PD	—	—	Nonrandomized, uncontrolled	Auto BM‐MSCs	n/a	14.76 ± 7.56	Intracerebral, sublateral ventricular zone	Safety; modest clinical improvement (UPDRS in “on” and “off” periods)	137
SCI	NCT00695149	I/II	Nonrandomized, uncontrolled	Auto BM‐MSCs	3–5 × 10^7^	<3 wk	Intrathecal	Safety (CESCT); motor improvement (4/5), stabilized condition (1/5)	139
SCI	—		Nonrandomized, uncontrolled	Auto BM‐MSCs	1 × 10^6^/kg body weight	<6 mo; >6 mo	Intrathecal	Safety; improved scores of BI (5/30)	138
SCI	NCT01274975	I	Nonrandomized, uncontrolled	Auto AT‐MSCs	4 × 10^8^	>12 mo	Intravenous	Some adverse effects, slightly improved scores of BI (1/8)	125
SCI	—	I/II	Nonrandomized, controlled	Auto BM‐MSCs	0.7‐1.2 × 10^6^	14‐43 days	Intrathecal	Safety; marked improvement in study group in ASIA scale from A to C (5/11) in comparison to control group (3/20)	142
SCI	—	—	Nonrandomized, controlled	Auto BM‐MSCs	5‐10 × 10^6^/kg body weight	3.6 ± 2.5 yr	1‐6° intrathecal (monthly for 6 mo)	Several side effects; improvement in ASIA scale A patients (12/40)	124
SCI	—	—	Nonrandomized, uncontrolled	Auto BM‐MSCs	1° 1‐4 × 10^6^/kg body weight 2–3° 1‐2 × 10^6^/kg body weight	3‐132 mo	1° at injury site 2–3° intrathecal (up to 21 days after 1°)	Safety; sensory (2/13) and motor functions (1/13) recovery	140
SCI	—	—	Nonrandomized, uncontrolled	Auto BM‐MSCs	1° 8 × 10^6^ 2° 4 × 10^7^ 3° 5 × 10^7^	Undisclosed	1° at injury site 2° intradural space (up to 21 days after 1°) 3° intrathecal (4‐8 wk after 1 and 2°)	Safety; significant improvement in motor and sensory functional recovery (6/10)	126
SCI	—	—	Case report	Auto 1° BMNCs 2° BMNCs 3° BM‐MSCs	1° 3.2 × 10^9^ 2° 5 × 10^8^ 3‐7° 1.3‐3.65 × 10^7^	3 mo	1° intravenous 2° intrathecal 3‐7° intrathecal (every 3‐4 mo)	Safety; improvement in ASIA scale A to C/D; sensation level decreased from Th1 to L3‐4; restored trunk control and bladder filling and control; restoration of the spinal cord continuity; improved muscle strength	128
MS	NCT01228266	II	Randomized, double‐blind, placebo‐controlled, crossover	Auto BM‐MSCs	1‐2 × 10^6^/kg body weight	2–10 yr	Intravenous	Safety; trend to lower cumulative number of gadolinium‐enhancing lesions on MRI; nonsignificant decrease of Th1 and modest decrease in Th17 lymphocytes population in peripheral blood	129
MS	NCT00395200	IIa	Nonrandomized, uncontrolled	Auto BM‐MSCs	1.1‐2 × 10^6^/kg body weight	5‐26 yr	Intravenous	Safety; improved visual acuity, contrast sensitivity and visual evoked response latency, increase in optic nerve area	130
MS	—	IIa	Nonrandomized, uncontrolled	Auto BM‐MSCs	Mean: 29.5 × 10^6^	8.6 ± 2.4 yr	Intrathecal	Safety; slight adverse postinjection events due to lumbar puncture; stability (16/22) and improvement (3/22) in the course of the disease measured by EDSS score; halting of T2 lesions development on MRI	131
DRE	NCT02497443	I	Randomized, controlled	Auto 1° BM‐MSCs 2° neural‐primed BM‐MSCs	1° 4–10 × 10^7^ 2° 2.7‐8 × 10^6^	15‐32 yr	1° intravenous 2° intrathecal	Safety; significant endpoint improvement vs AEDs only group in monthly seizure frequency, NHS seizure severity score and anxiety score	141
DRE	—	—	Nonrandomized, uncontrolled	Auto 1° BMNCs 2° BMNCs 3‐6° BM‐MSCs	1° 0.38‐1.72 × 10^9^ 2° 0.5 × 10^9^ 3‐6° 18.5‐40 × 10^6^	8 mo‐6 yr	1° intravenous 2° intrathecal 3‐6° intrathecal	Safety; neurological and cognitive improvement in all patients; reduction in the number of epileptic seizures; absence of status epilepticus episodes; improved motor functions	132

Abbreviations: AEDs, antiepileptic drugs; allo, allogenic; ASIA, American Spinal Injury Association; AT‐MSCs, adipose tissue mesenchymal stem cells; Auto, autologous; BI, Barthel index; BM‐MSCs, bone marrow mesenchymal stem cells; BMNCs, bone marrow nucleated cells; CESCT, Committee on Effectiveness and Safety of Clinical Treatment; CP, cerebral palsy; CTCAE, Common Terminology Criteria for Adverse Events; DRE, drug‐resistant epilepsy; EDSS, expanded disability status scale; ESS, European Stroke Scale; F‐M, Fugl‐Meyer; GMFCS, Gross Motor Function Classification System; L, lumbar region; MRI, magnetic resonance imaging; mRS, modified Rankin scale; MS, multiple sclerosis; NHS, National Health Service; NIHSS, National Institutes of Health Stroke Scale; PD, Parkinson's disease; PVS, persistent vegetative state; SCI, spinal cord injury; TBI, traumatic brain injury; Th, thoracic region; UPDRS, Unified Parkinson's disease rating scale.

The majority of clinical trials have shown the safety of a variety of MSC applications. Severe side effects were noted when cells were transplanted during stereotactic surgery in TBI patients.^123^ Nevertheless, most adverse events were correlated with the conducted procedure, and none of them were followed with sequelae. Side effects (spasticity, neuropathic pain, and encephalomyelitis) were also observed in a trial performed by Kishk et al., although the American Spinal Injury Association (ASIA) scales of the patients were rated down from type A to type B.^124^ In a study by Ra et al., the authors reported adverse effects following transplantation in all patients enrolled in the study, although their electrophysiological (somatosensory evoked potential [SSEP] and motor evoked potential [MEP]) recordings were not significantly different than before implantation.^125^ In most of the studies, the safety and efficiency of therapies were assessed additionally by magnetic resonance imaging (MRI).^110^, ^124^, ^125^, ^126^, ^127^, ^128^, ^129^, ^130^, ^131^, ^132^, ^133^, ^134^, ^135^, ^136^, ^137^, ^138^, ^139^ No significant anatomical, structural, or parenchymal abnormalities or tumor formation were observed.

In most of the studies, bacterial tests were performed.^123^, ^125^, ^127^, ^130^, ^133^, ^134^, ^135^, ^136^, ^137^, ^138^, ^140^, ^141^ In the majority of studies, karyotype analysis was also carried out.^123^, ^125^, ^128^, ^130^, ^132^, ^136^, ^137^, ^138^, ^140^ Additionally, the differentiation potential of MSCs was assessed in a few clinical studies.^125^, ^130^, ^131^, ^136^, ^138^, ^140^, ^142^ Only in a minority of studies parallel testing in animals was conducted.^143^, ^144^ In some clinical trials, safety evaluation was performed, proving the lack of tumorigenicity of MSCs after administration to immunodeficient mice via subcutaneous or intraspinal transplantations.^125^, ^136^

Transplantation of MSCs resulted in neurologic improvement in the majority of clinical trials. Superior performance in activities of daily living (in TBI, stroke, and SCI) and motor recovery (in TBI, stroke, CP, and SCI) was noted. In clinical studies in groups of patients with SCI, significant improvements were observed in sensory level and motor function, as well as in general outcome with the ASIA scale.^124^, ^128^, ^142^ Tian et al. demonstrated that in TBI patients in a vegetative state, consciousness improved after MSC transplantation.^145^ In patients with MS, a tendency toward a decline in active inflammation processes and stabilization of disease progression was observed.^130^, ^131^ An enormous improvement has been noted in patients diagnosed with DRE, in whom transplantation of autologous MSCs resulted in amelioration of epileptic incidences.^132^ This suggests a profound role of MSCs in the repair of epileptic brains.^132^, ^141^ Modest outcomes have been noted in clinical trials with PD patients, in whom only slight improvements in the Unified Parkinson's disease rating scale scale were obtained. However, most patients claimed subjective marginal improvement of symptoms.^137^

In several studies, slight changes on neuroimaging were noted. Steinberg et al. reported signal changes on T2 fluid attenuation inversion recovery (FLAIR) MRI (13/18) and in the number of contrast‐enhancing areas (15/18) in stroke patients 1 or 2 weeks after transplantation.^110^ Moreover, they found a significant correlation between these changes and clinical outcomes at the 12‐month follow‐up.

Slight changes on MRI were observed in a study performed by Bang et al. Researchers noticed less prominent atrophic changes following stroke in the MSC‐treated group than in the control group.^133^

Honmou et al. found a reduction in infarct size on FLAIR MRI in 7 out of 12 stroke patients after treatment.^135^ However, because of the lack of a control group in this study, the authors cannot exclude spontaneous recovery as a cause for changes in infarct size. Nevertheless, they also noted a significant correlation between neuroimaging results and mean changes in the National Institutes of Health Stroke Scale score.

In a study by Lee et al., researchers noted a correlation between clinical improvements of stroke patients and SVZ damage defined by diffusion MRI.^134^ They noticed a relationship between less SVZ damage and clinical improvement in the MSC‐treated group of patients but did not see this relationship in the rest of the groups (MSC‐treated patients with more SVZ damage and corresponding controls). Moreover, they determined SDF‐1α levels in patient plasma at the time of first transplantation. Their analysis revealed a significant correlation between SDF‐1α plasma levels and patient outcome, defined as scores on the Barthel index and modified Rankin scale.

Currently, there are two ongoing clinical trials concerning the application of autologous MSCs in stroke, one of which is a randomized, controlled, and observer‐blinded trial. The detailed methodology of these studies has already been published.^146^, ^147^

In clinical trials concerning patients with SCI, Park and colleagues reported for the first time improvement on electrophysiological results, assessed by SSEP and MEP recordings, and MRI examination.^148^ This improvement may be related to several factors. Direct administration of MSCs into the site of injury is a more effective method for SCI recovery than intrathecal injection. On the other hand, three patients from the group who showed motor recovery had an incomplete SCI with residual neurological function.

In a study by Jarocha et al. concerning one patient with total SC interruption at the Th2‐3 level, muscle strength at the left lower extremity improved from plegia to deep paresis (1° on the Lovett scale). Moreover, the ability to move her lower extremities against gravity supported by the movements in her quadriceps was restored. Neurophysiologic examination including electromyography, electroneurography SEP, and MEP recordings objectively confirmed the improvement. Moreover, MRI demonstrated restoration of spinal cord continuity.^128^

In a study concerning the application of MSCs in MS, Bonab et al. showed that one intrathecal injection of autologous MSCs in patients with secondary progressive MS resulted in stabilization of MRI findings in approximately 70% of participants. As the authors underlined, these results are very appealing in comparison to those gained from standard pharmacological treatments with interferon beta‐1a and mitoxantrone.^131^ MRI studies were also performed to separate the “honey‐moon effect” from the real effect of the therapy. Interestingly, the results obtained by Bonab et al. showed that 1 year after transplantation of cells, new lesions started to appear on MRI but without clinical manifestations. This observation may be caused by the diminishing effect of therapy with time and stresses the necessity of repeated transplantations.

In the study by Connick et al., researchers proposed a new approach to measure therapeutic outcome after single intravenous administration of autologous MSCs by assessing functioning of the anterior visual pathway.^130^ They showed that visual evoked response latency and an increase in optic nerve area appeared as a result of the improvement in visual acuity from the applied therapy. Despite the important conclusions from the above clinical trials, the lack of a control group of patients is a major drawback of these studies.

In fact, in a randomized placebo‐controlled phase II trial performed by Llufriu et al., therapeutic effects were seen in only groups treated with MSCs.^129^ Compared with the placebo group, the MSC‐treated group had changes in the mean cumulative number of gadolinium‐enhancing lesions on MRI and a tendency for lower numbers of Th1 lymphocytes in blood; however, none of the differences was statistically significant. These observations underline the importance of controlled studies with larger groups of enrolled patients, especially for testing MSC therapies in diseases with unstable clinical performance, as is the case for MS.

Only some studies have implemented repeated transplantation of MSCs.^123^, ^126^, ^128^, ^132^, ^140^, ^141^ In other studies, deterioration of the clinical status of patients was noted, which may have been due to the single MSC administration.^131^

As shown above, MSCs ameliorate functional deficits in several central nervous system diseases in both experimental animal models and in the clinic. Therapeutic mechanisms may include neuroprotective effects, immunomodulation, tissue remodeling, and activation of local progenitors. Therefore, MSCs prepare the environment for axonal ingrowth, stimulate angiogenesis, and result in functional recovery.

## 5 DISCUSSION AND FUTURE REMARKS

Growing knowledge about MSC regenerative potential raises great hopes for applying them in the clinic. However, there is still space for improvement, and more preclinical studies have to be performed to evaluate cell culture conditions, the potential for neuronal priming, and the timing and route of administration to obtain the best improvements for patients.

First, there is a lack of consistent data showing the optimal source of MSCs for transplantation. As shown in basic studies, MSCs can differ between sources in their regenerative potential, as shown by, for example, the different levels of secreted trophic factors or the propensity of cells to differentiate toward different lineages. However, there are many discrepancies between published data on the properties of BM‐, AT‐, and WJ‐MSCs. Therefore, a comprehensive study is needed to obtain reliable results. Such inquiry could also improve cell preparation methods for clinical trials and define optimal cell culture conditions. Another issue is the reported differences between MSCs from healthy donors and MSCs from diseased patients that could influence the final outcomes of therapies.^44^ More detailed studies are also needed to obtain a consensus on this phenomenon.

Second, similar numbers of transplanted cells have led to improved clinical outcomes in neurological disorders. However, there is a lack of comprehensive studies discussing these issues by evaluating different doses in one clinical study.

Another issue is a lack of consensus for the best MSC transplantation method. Intravascularly transplanted MSCs may become trapped in the lungs and liver, from which they can release microvesicles and immunomodulative factors.^56^ In some preclinical studies, such entrapment resulted in serious side effects, such as increased risk for iatrogenic atelectasis and lethal pulmonary thromboembolism; however, such observations were made when MSCs were transplanted into healthy animals.^149^ In those animals, active migration of MSCs cannot be enhanced, so the cells inertly accumulate in the lungs. In many studies (in stroke and MS), intravascular administration of MSCs despite their presence near the injury area has a significant influence on outcome after treatment.^129^, ^130^, ^133^ This suggests that MSCs can influence inflammation in the brain through the peripheral circulatory system. However, the residual presence of MSCs can also be caused by their migration and poor survival in the nervous system. In contrast, studies have shown the presence of MSCs in the nervous system for up to 8 weeks, which could be long enough to exert therapeutic effects.^119^

Invasive ways of transplantation of MSCs can provoke many side effects and potentially enhance inflammation in the nervous system, which can (a) cause additional changes in the nervous system and/or (b) diminish therapeutic potential. Therefore, intrathecal implementation of MSCs can transplant them directly to cerebrospinal fluid without any significant adverse effects. On the other hand, SCI clinical trials have shown that the best results occur when MSCs are transplanted directly into damaged tissue. However, our clinical trials have shown that intrathecal implementation can also be very successful.^128^, ^132^ Therefore, the combination of intrathecal and intravenous administration can be considered an effective way to modulate the environment inside the nervous system as well as the adaptive immune response. Importantly, both of these methods of transplantation can be used for repeated injections.

As shown in clinical and preclinical studies, the mainstays of MSC regenerative potential are the capabilities for immunomodulation and secretion of many trophic factors. Therefore, multiple injections can provide continuous stimulation for repair.^123^, ^126^, ^128^, ^132^, ^140^, ^141^ Several clinical trials have shown that repeated transplantations of cells are beneficial for patient outcome, which necessitates the standardization of a reasonable cell injection strategy. In our hands, such a method seems to be intrathecal injection, which is also popular in other studies.^128^, ^132^ Although some side effects may appear, there is no danger to patient health or life. Moreover, this route of administration has undoubted advantages in being transplanted directly to the nervous system, allowing the cells to easily migrate to regions of degeneration. Nonetheless, because some studies reported complications after this type of administration, such an approach still remains to be evaluated. Less invasive methods of MSC transplantation might be intranasal injections; however, to date, this method of injection has only been evaluated in clinical studies for drug administration.^150^

As mentioned above, the trophic character of MSCs underlies clinical improvement in patients. It was also shown in preclinical studies that MSCs are present up to weeks after transplantation, and this time is sufficient for promotion of neurogenesis in adult mice with HD.^151^ The issue, which fortunately is being evaluated, is whether MSCs in fact can engraft and differentiate into neurons and replace the lost ones. As studied in MSCs overexpressing Ngn‐1, modified MSCs have enhanced electrophysiological characteristics, and some of them show neuronal‐like characteristics after transplantation. Nevertheless, it cannot be claimed that MSCs transdifferentiate into neurons. Additionally, the cells disappear after 8 weeks. To date, no study has performed electrophysiological tests on transplanted MSCs with neuronal‐like characteristics. Though studies have claimed that modification of MSCs or priming can enhance neuronal‐like features, calling those cells neurons, without a proper battery of tests, is incorrect.

Another emerging issue is related to the necessity of side‐to‐side clinical and preclinical studies. Such an approach has been implemented in only a minority of clinical studies.^143^, ^144^ This type of scientific approach is of necessary to not only identify potential improvements but also ensure solid scientific methods are being employed to explain observed phenomena.

It is also worth mentioning that the discrepancy between preclinical and clinical trials can be caused by the selection of patients in clinical trials. Unfortunately, because there are still not well‐standardized procedures concerning MSC transplantation, patients enrolled in clinical trials are usually incurable by every other common method or their disorder has lasted for a long time. The abovementioned reasons can diminish the clinical outcomes in patients in clinical trials in comparison to those seen in preclinical trials. This also addresses the problem of the best therapeutic window. It was shown in vast of preclinical studies that MSC transplantation shortly after injury is most promising for obtaining the greatest therapeutic effect.^152^, ^153^ This is especially crucial for neurological disorders such as stroke, TBI, SE or SCI, in which there is no time to obtain and expand autologous MSCs from patients. Currently, this approach is not possible in clinical trials. In the future, cell banks can store ready‐made products to be transplanted in an allogeneic manner.

It has been shown in preclinical studies that one of the ways to enhance the positive impact of MSCs on the repair of the nervous system is to modify them with trophic factors. For safety reasons, allogeneic transplantations will be the first choice. In other cases, in vitro priming of autologous MSCs can also be efficient, which has been shown in CP clinical trials.^136^ Detailed in vitro studies combined with in vivo testing are needed to evaluate standardized protocols of such priming/modification strategies. The effect of modification must be profound to risk transplantation of genetically modified cells in clinical studies. However, as shown in studies using the stable SB623 cell line, the usage of such cells can be safe and may increase the therapeutic potential of MSCs.^106^, ^109^, ^110^

## 6 CONCLUSIONS

**FIGURE 2:**
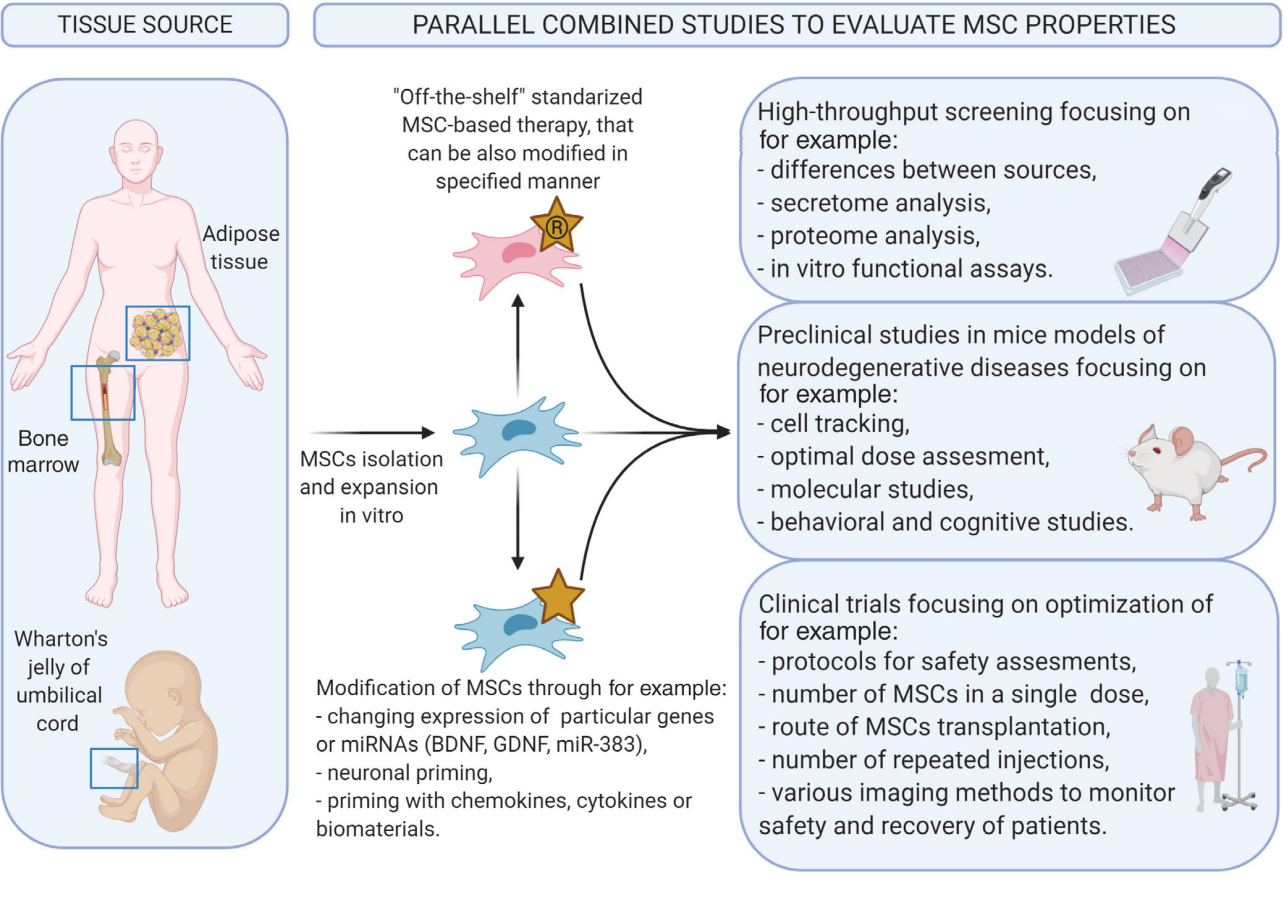
Schematic overview of the most essential aspects of mesenchymal stem cell (MSC)‐based cell therapies in treatment of nervous system diseases. Created with BioRender.com

Published results of MSC transplantation in various neurological diseases, especially in clinical trials, show that MSCs have great potential to improve patient symptoms and quality of life, whereas traditional medicine offers no efficient treatment. However, the design of experiments within clinical studies and preclinical studies leaves vast space for reasonable criticism of the regenerative potential of MSCs. Therefore, more consistent evaluation of MSCs is needed. Taking into consideration the multimodal characteristics of MSCs, studies evaluating their role in repair should also consider such characteristics. More advanced and parallel methods should be involved, such as proteome and secretome studies, in vitro and in vivo functional studies and strictly controlled preclinical studies (Figure 2). As it is difficult to implement a placebo control in clinical trials, especially in pediatric diseases, there is a great need to perform detailed studies in preclinical trials involving advanced methodology and focusing on a deep understanding of the mechanism of the therapeutic potential of MSCs. Therefore, multidisciplinary studies involving clinicians and scientists from various specialties can pave a more reliable way for introducing MSCs into the clinic. We hope that our review uniformly summarizes both the promise and potential routes of advancement of MSCs that can provide information for consideration by clinicians planning future clinical trials.

## CONFLICT OF INTEREST

The authors declared no potential conflicts of interest.

### AUTHOR CONTRIBUTIONS

B.B., M.S., O.M., and M.M.: manuscript writing, final approval of the manuscript.

## Data Availability

Data sharing is not applicable to this article as no new data were created or analyzed in this study.

## References

[1] Fu H , Hardy J , Duff KE . Selective vulnerability in neurodegenerative diseases. Nat Neurosci. 2018;21(10):1350‐1358. 30250262 10.1038/s41593-018-0221-2 PMC6360529

[2] Mortezaee K , Khanlarkhani N , Beyer C , Zendedel A . Inflammasome: its role in traumatic brain and spinal cord injury. J Cell Physiol. 2018;233(7):5160‐5169. 29150951 10.1002/jcp.26287

[3] Lingam I , Robertson NJ . Magnesium as a neuroprotective agent: a review of its use in the fetus, term infant with neonatal encephalopathy, and the adult stroke patient. Dev Neurosci. 2018;40(1):1‐12. 29408814 10.1159/000484891

[4] Pierce AA , Xu AW . De novo neurogenesis in adult hypothalamus as a compensatory mechanism to regulate energy balance. J Neurosci. 2010;30(2):723‐730. 20071537 10.1523/JNEUROSCI.2479-09.2010 PMC3080014

[5] Louveau A , Herz J , Alme MN , et al. CNS lymphatic drainage and neuroinflammation are regulated by meningeal lymphatic vasculature. Nat Neurosci. 2018;21(10):1380‐1391. 30224810 10.1038/s41593-018-0227-9 PMC6214619

[6] Castorina A , Szychlinska M , Marzagalli R , Musumeci G . Mesenchymal stem cells‐based therapy as a potential treatment in neurodegenerative disorders: is the escape from senescence an answer? Neural Regen Res. 2015;10(6):850‐858. 26199588 10.4103/1673-5374.158352 PMC4498333

[7] Lindvall O . Human fetal dopamine neurons grafted into the striatum in two patients with severe Parkinson's disease. Arch Neurol. 1989;46(6):615‐631. 2786405 10.1001/archneur.1989.00520420033021

[8] Barker R , Drouin‐Ouellet J , Parmar M . Cell‐based therapies for Parkinson disease—past insights and future potential. Nat Rev Neurol. 2015;11(9):492‐503. 26240036 10.1038/nrneurol.2015.123

[9] Peruzzotti‐Jametti L , Bernstock JD , Vicario N , et al. Macrophage‐derived extracellular succinate licenses neural stem cells to suppress chronic neuroinflammation. Cell Stem Cell. 2018;22(3):355‐368.e13. 29478844 10.1016/j.stem.2018.01.020 PMC5842147

[10] Olanow CW , Goetz CG , Kordower JH , et al. A double‐blind controlled trial of bilateral fetal nigral transplantation in Parkinson's disease. Ann Neurol. 2003;54(3):403‐414. 12953276 10.1002/ana.10720

[11] Takahashi K , Tanabe K , Ohnuki M , et al. Induction of pluripotent stem cells from adult human fibroblasts by defined factors. Cell. 2007;131(5):861‐872. 18035408 10.1016/j.cell.2007.11.019

[12] Bunpetch V , Wu H , Zhang S , Ouyang H . From “bench to bedside”: current advancement on large‐scale production of mesenchymal stem cells. Stem Cells Dev. 2017;26(22):1662‐1673. 28934885 10.1089/scd.2017.0104

[13] Gao F , Chiu SM , Motan DAL , et al. Mesenchymal stem cells and immunomodulation: current status and future prospects. Cell Death Dis. 2016;7:e2062. 26794657 10.1038/cddis.2015.327 PMC4816164

[14] Salgado AJ , Sousa JC , Costa BM , et al. Mesenchymal stem cells secretome as a modulator of the neurogenic niche: basic insights and therapeutic opportunities. Front Cell Neurosci. 2015;9:249. 10.3389/fncel.2015.00249 PMC4499760 26217178

[15] Friedenstein AJ , Chailkhjan RK , Lalykina KS . The development of fibroblast colonies in monolayer cultures of Guinea‐pig bone marrow and spleen cells. Cell Tissue Kinet. 1970;3:393‐403. 5523063 10.1111/j.1365-2184.1970.tb00347.x

[16] Jackson W , Aragon A , Djouad F . Mesenchymal progenitor cells derived from traumatized human muscle. J Tissue Eng Regen Med. 2009;3(2):129‐138. 19170141 10.1002/term.149 PMC2814161

[17] Zuk PA , Zhu M , Mizuno H , et al. Multilineage cells from human adipose tissue: implications for cell‐based therapies. Tissue Eng. 2001;7(2):211‐228. 11304456 10.1089/107632701300062859

[18] Erices A , Conget P , Minguell JJ . Mesenchymal progenitor cells in human umbilical cord blood. Br J Haematol. 2000;109(1):235‐242. 10848804 10.1046/j.1365-2141.2000.01986.x

[19] Deuse T , Stubbendorff M , Tang‐Quan K , et al. Immunogenicity and immunomodulatory properties of umbilical cord lining mesenchymal stem cells. Cell Transplant. 2011;20(5):655‐667. 21054940 10.3727/096368910X536473

[20] Romanov YA . Searching for alternative sources of postnatal human mesenchymal stem cells: candidate MSC‐like cells from umbilical cord. Stem Cells. 2003;21(1):105‐110. 12529557 10.1634/stemcells.21-1-105

[21] Sarugaser R , Lickorish D , Baksh D , Hosseini MM , Davies JE . Human umbilical cord perivascular (HUCPV) cells: a source of mesenchymal progenitors. Stem Cells. 2005;23(2):220‐229. 15671145 10.1634/stemcells.2004-0166

[22] Wang H‐S , Hung S‐C , Peng S‐T , et al. Mesenchymal stem cells in the Wharton's jelly of the human umbilical cord. Stem Cells. 2004;22(7):1330‐1337. 15579650 10.1634/stemcells.2004-0013

[23] Gronthos S , Mankani M , Brahim J , Robey PG , Shi S . Postnatal human dental pulp stem cells (DPSCs) in vitro and in vivo. Proc Natl Acad Sci U S A. 2000;97(25):13625‐13630. 11087820 10.1073/pnas.240309797 PMC17626

[24] Jones EA , English A , Henshaw K , et al. Enumeration and phenotypic characterization of synovial fluid multipotential mesenchymal progenitor cells in inflammatory and degenerative arthritis. Arthritis Rheum. 2004;50(3):817‐827. 15022324 10.1002/art.20203

[25] De Bari C , Dell'Accio F , Tylzanowski P , et al. Multipotent mesenchymal stem cells from adult human synovial membrane. Arthritis Rheum. 2001;44(8):1928‐1942. 11508446 10.1002/1529-0131(200108)44:8<1928::AID-ART331>3.0.CO;2-P

[26] Tsai MS , Lee JL , Chang YJ , Hwang SM . Isolation of human multipotent mesenchymal stem cells from second‐trimester amniotic fluid using a novel two‐stage culture protocol. Hum Reprod. 2004;19(6):1450‐1456. 15105397 10.1093/humrep/deh279

[27] Campagnoli C , Roberts IAG , Kumar S , Bennett PR , Bellantuono I , Fisk NM . Identification of mesenchymal stem/progenitor cells in human first‐trimester fetal blood, liver, and bone marrow. Blood. 2001;98(8):2396‐2402. 11588036 10.1182/blood.v98.8.2396

[28] Bharadwaj S , Liu G , Shi Y , et al. Multipotential differentiation of human urine‐derived stem cells: potential for therapeutic applications in urology. Stem Cells. 2013;31(9):1840‐1856. 23666768 10.1002/stem.1424

[29] Gargett CE , Ye L . Endometrial reconstruction from stem cells. Fertil Steril. 2012;98(1):11‐20. 22657248 10.1016/j.fertnstert.2012.05.004

[30] Lojewski X , Srimasorn S , Rauh J , et al. Perivascular mesenchymal stem cells from the adult human brain harbor no instrinsic neuroectodermal but high mesodermal differentiation potential. Stem Cells Translational Medicine. 2015;4(10):1223‐1233. 26304036 10.5966/sctm.2015-0057 PMC4572903

[31] Dominici M , Le BK , Mueller I , et al. Minimal criteria for defining multipotent mesenchymal stromal cells. The International Society for Cellular Therapy position statement. Cytotherapy. 2006;8(4):315‐317. 16923606 10.1080/14653240600855905

[32] Tse WT , Pendleton JD , Beyer WM , Egalka MC , Guinan EC . Suppression of allogeneic T‐cell proliferation by human marrow stromal cells: implications in transplantation. Transplantation. 2003;75(3):389‐397. 12589164 10.1097/01.TP.0000045055.63901.A9

[33] Najar M , Raicevic G , Kazan HF , et al. Immune‐related antigens, surface molecules and regulatory factors in human‐derived mesenchymal stromal cells: the expression and impact of inflammatory priming. Stem Cell Rev Rep. 2012;8(4):1188‐1198. 22983809 10.1007/s12015-012-9408-1

[34] Catacchio I , Berardi S , Reale A , et al. Evidence for bone marrow adult stem cell plasticity: properties, molecular mechanisms, negative aspects, and clinical applications of hematopoietic and mesenchymal stem cells transdifferentiation. Stem Cells Int. 2013;2013:1‐11. 10.1155/2013/589139 PMC3625599 23606860

[35] Gnecchi M , Danieli P , Malpasso G , et al. Paracrine mechanisms of mesenchymal stem cells in tissue repair. Methods Mol Biol. 2016;1416:123‐146. 27236669 10.1007/978-1-4939-3584-0_7

[36] Teng YD . Functional multipotency of stem cells and recovery neurobiology of injured spinal cords. Cell Transplant. 2019;28(4):451‐459. 31134830 10.1177/0963689719850088 PMC6628559

[37] Ropper AE , Thakor DK , Han I , et al. Defining recovery neurobiology of injured spinal cord by synthetic matrix‐assisted hMSC implantation. Proc Natl Acad Sci. 2017;114(5):E820‐E829. 28096400 10.1073/pnas.1616340114 PMC5293074

[38] Amable P , Teixeira MV , Carias RB , Granjeiro J , Borojevic R . Protein synthesis and secretion in human mesenchymal cells derived from bone marrow, adipose tissue and Wharton's jelly. Stem Cell Res Ther. 2014;5(2):53. 24739658 10.1186/scrt442 PMC4055160

[39] Amable PR , Teixeira MVT , Carias RBV , Granjeiro JM , Borojevic R . Mesenchymal stromal cell proliferation, gene expression and protein production in human platelet‐rich plasma‐supplemented media. PLoS One. 2014;9(8):e104662. 25115920 10.1371/journal.pone.0104662 PMC4130592

[40] Amable PR , Teixeira MVT , Carias RBV , Granjeiro JM , Borojevic R . Gene expression and protein secretion during human mesenchymal cell differentiation into adipogenic cells. BMC Cell Biol. 2014;15:46. 25526965 10.1186/s12860-014-0046-0 PMC4293810

[41] Fuchs E , Tumbar T , Guasch G . Socializing with the neighbors: stem cells and their niche. Cell. 2004;116(6):769‐778. 15035980 10.1016/s0092-8674(04)00255-7

[42] Elahi KC , Klein G , Avci‐Adali M , Sievert KD , MacNeil S , Aicher WK . Human mesenchymal stromal cells from different sources diverge in their expression of cell surface proteins and display distinct differentiation patterns. Stem Cells Int. 2016;2016:1‐9. 10.1155/2016/5646384 PMC4684891 26770208

[43] Salehinejad P , Alitheen NB , Ali AM , et al. Comparison of different methods for the isolation of mesenchymal stem cells from human umbilical cord Wharton's jelly. In Vitro Cell Dev Biol Anim. 2012;48(2):75‐83. 22274909 10.1007/s11626-011-9480-x

[44] Koh SH , Baik W , Noh MY , et al. The functional deficiency of bone marrow mesenchymal stromal cells in ALS patients is proportional to disease progression rate. Exp Neurol. 2012;233(1):472‐480. 22119626 10.1016/j.expneurol.2011.11.021

[45] Fazzina R , Iudicone P , Fioravanti D , et al. Potency testing of mesenchymal stromal cell growth expanded in human platelet lysate from different human tissues. Stem Cell Res Ther. 2016;7(1):122. 27557940 10.1186/s13287-016-0383-3 PMC4997686

[46] Jin H , Bae Y , Kim M , et al. Comparative analysis of human mesenchymal stem cells from bone marrow, adipose tissue, and umbilical cord blood as sources of cell therapy. Int J Mol Sci. 2013;14(9):17986‐18001. 24005862 10.3390/ijms140917986 PMC3794764

[47] Nasir GA , Mohsin S , Khan M , et al. Mesenchymal stem cells and interleukin‐6 attenuate liver fibrosis in mice. J Transl Med. 2013;11:78. 23531302 10.1186/1479-5876-11-78 PMC3636128

[48] Ayatollahi M , Talaei‐Khozani T , Razmkhah M . Growth suppression effect of human mesenchymal stem cells from bone marrow, adipose tissue, and Wharton's jelly of umbilical cord on PBMCs. Iran J Basic Med Sci. 2016;19(2):145‐153. 27081458 PMC4818361

[49] Karaöz E , Çetinalp Demircan P , Erman G , Güngörürler E , Eker Sarıboyacı A . Comparative analyses of immune‐suppressive characteristics of bone‐marrow, Wharton's jelly a nd adipose‐tissue derived human MSCs. Turk J Hematol. 2017:34(3):213–225. 10.4274/tjh.2016.0171 PMC5544040 27610554

[50] Teixeira FG , Carvalho MM , Panchalingam KM , et al. Impact of the Secretome of human mesenchymal stem cells on brain structure and animal behavior in a rat model of Parkinson's disease. Stem Cells Translational Med. 2017;6(2):634‐646. 10.5966/sctm.2016-0071 PMC5442797 28191785

[51] Balasubramanian S , Thej C , Venugopal P , et al. Higher propensity of Wharton's jelly derived mesenchymal stromal cells towards neuronal lineage in comparison to those derived from adipose and bone marrow. Cell Biol Int. 2013;37(5):507‐515. 23418097 10.1002/cbin.10056

[52] Lee T . Stem cell therapy independent of stemness. World J Stem Cells. 2012;4(12):120‐124. 23516128 10.4252/wjsc.v4.i12.120 PMC3600562

[53] Yang CC , Shih YH , Ko MH , Hsu SY , Cheng H , Fu YS . Transplantation of human umbilical mesenchymal stem cells from Wharton's jelly after complete transection of the rat spinal cord. PLoS One. 2008;3(10):e3336. 18852872 10.1371/journal.pone.0003336 PMC2566594

[54] Sohni A , Verfaillie CM . Mesenchymal stem cells migration homing and tracking. Stem Cells Int. 2013;2013:1‐8. 10.1155/2013/130763 PMC3806396 24194766

[55] Lee NK , Kim HS , Yoo D , et al. Magnetic resonance imaging of ferumoxytol‐labeled human mesenchymal stem cells in the mouse brain. Stem Cell Rev Rep. 2017;13(1):127‐138. 27757917 10.1007/s12015-016-9694-0 PMC5346117

[56] Agadi S , Shetty AK . Concise review: prospects of bone marrow mononuclear cells and mesenchymal stem cells for treating status epilepticus and chronic epilepsy. Stem Cells. 2015;33(7):2093‐2103. 25851047 10.1002/stem.2029 PMC7023904

[57] Bang OY , Moon GJ , Kim DH , et al. Stroke induces mesenchymal stem cell migration to infarcted brain areas via CXCR4 and C‐met signaling. Transl Stroke Res. 2017;8(5):449‐460. 10.1007/s12975-017-0538-2 28547726

[58] Lee SH , Jin KS , Bang OY , et al. Differential migration of mesenchymal stem cells to ischemic regions after middle cerebral artery occlusion in rats. PLoS One. 2015;10(8):1‐11. 10.1371/journal.pone.0134920 PMC4524688 26241653

[59] Park S , Jang H , Kim BS , et al. Directional migration of mesenchymal stem cells under an SDF‐1α gradient on a microfluidic device. PLoS One. 2017;12(9):1‐18. 10.1371/journal.pone.0184595 PMC5590985 28886159

[60] Ma S , Xie N , Li W , Yuan B , Shi Y , Wang Y . Immunobiology of mesenchymal stem cells. Cell Death Differ. 2014;21(2):216‐225. 24185619 10.1038/cdd.2013.158 PMC3890955

[61] Di TM , Bassi G , Ricciardi M , et al. Comparative study of immune regulatory properties of stem cells derived from different tissues. Stem Cells Dev. 2013;22(22):2990‐3002. 23819720 10.1089/scd.2013.0204 PMC3840473

[62] Loane DJ , Kumar A . Microglia in the TBI brain: the good, the bad, and the dysregulated. Exp Neurol. 2016;275:316‐327. 26342753 10.1016/j.expneurol.2015.08.018 PMC4689601

[63] Simon DW , McGeachy MJ , Bayır H , et al. The far‐reaching scope of neuroinflammation after traumatic brain injury. Nat Rev Neurol. 2017;13(3):171‐191. 28186177 10.1038/nrneurol.2017.13 PMC5675525

[64] Ziebell JM , Morganti‐Kossmann MC . Involvement of pro‐ and anti‐inflammatory cytokines and chemokines in the pathophysiology of traumatic brain injury. Neurotherapeutics. 2010;7(1):22‐30. 20129494 10.1016/j.nurt.2009.10.016 PMC5084109

[65] Obermeier B , Daneman R , Ransohoff RM . Development, maintenance and disruption of the blood‐brain barrier. Nat Med. 2013;19(12):1584‐1596. 24309662 10.1038/nm.3407 PMC4080800

[66] Lucassen PJ , Oomen CA , Naninck EFG , et al. Regulation of adult neurogenesis and plasticity by (early) stress, glucocorticoids, and inflammation. Cold Spring Harb Perspect Biol. 2015;7(9):a021303. 26330520 10.1101/cshperspect.a021303 PMC4563706

[67] Zhang X , Zhang Q , Li W , et al. Therapeutic effect of human umbilical cord mesenchymal stem cells on neonatal rat hypoxic‐ischemic encephalopathy. J Neurosci Res. 2014;92(1):35‐45. 24265136 10.1002/jnr.23304

[68] Abrams MB , Dominguez C , Pernold K , et al. Multipotent mesenchymal stromal cells attenuate chronic inflammation and injury‐induced sensitivity to mechanical stimuli in experimental spinal cord injury. Restor Neurol Neurosci. 2009;27(4):307‐321. 19738324 10.3233/RNN-2009-0480

[69] Huang PY , Shih YH , jhan TY , et al. Xenograft of human umbilical mesenchymal stem cells from Wharton's jelly as a potential therapy for rat pilocarpine‐induced epilepsy. Brain Behav Immun. 2016;54:45‐58. 26732826 10.1016/j.bbi.2015.12.021

[70] Budoni M , Fierabracci A , Luciano R , Petrini S , di Ciommo V , Muraca M . The immunosuppressive effect of mesenchymal stromal cells on B lymphocytes is mediated by membrane vesicles. Cell Transplant. 2013;22(2):369‐379. 23433427 10.3727/096368911X582769

[71] Donega V , Nijboer CH , van Tilborg G , et al. Intranasally administered mesenchymal stem cells promote a regenerative niche for repair of neonatal ischemic brain injury. Exp Neurol. 2014;261:53‐64. 24945601 10.1016/j.expneurol.2014.06.009

[72] Nakajima H , Uchida K , Guerrero AR , et al. Transplantation of mesenchymal stem cells promotes an alternative pathway of macrophage activation and functional recovery after spinal cord injury. J Neurotrauma. 2012;29(8):1614‐1625. 22233298 10.1089/neu.2011.2109 PMC3353766

[73] Yang H , Xie Z , Wei L , et al. Human umbilical cord mesenchymal stem cell‐derived neuron‐like cells rescue memory deficits and reduce amyloid‐beta deposition in an AbetaPP/PS1 transgenic mouse model. Stem Cell Res Ther. 2013;4(4):76. 23826983 10.1186/scrt227 PMC3854736

[74] Naaldijk Y , Jäger C , Fabian C , et al. Effect of systemic transplantation of bone marrow‐derived mesenchymal stem cells on neuropathology markers in APP/PS1 Alzheimer mice. Neuropathol Appl Neurobiol. 2017;43:299‐314. 26918424 10.1111/nan.12319

[75] Ma T , Gong K , Ao Q , et al. Intracerebral transplantation of adipose‐derived mesenchymal stem cells alternatively activates microglia and ameliorates neuropathological deficits in Alzheimer's disease mice. Cell Transplant. 2013;22:113‐126. 10.3727/096368913X672181 24070198

[76] Menge T , Zhao Y , Zhao J , et al. Mesenchymal stem cells regulate blood brain barrier integrity in traumatic brain injury through production of the soluble factor TIMP3. Sci Transl Med. 2012;21(4161):161‐150. 10.1126/scitranslmed.3004660 PMC3890105 23175708

[77] Teixeira FG , Carvalho MM , Neves‐Carvalho A , et al. Secretome of mesenchymal progenitors from the umbilical cord acts as modulator of neural/glial proliferation and differentiation. Stem Cell Rev Rep. 2015;11(2):288‐297. 25420577 10.1007/s12015-014-9576-2

[78] Cao N , Liao T , Liu J , et al. Clinical‐grade human umbilical cord‐derived mesenchymal stem cells reverse cognitive aging via improving synaptic plasticity and endogenous neurogenesis. Cell Death Dis. 2017;8(8):e2996. 28796260 10.1038/cddis.2017.316 PMC5596535

[79] Li Y , Chen J , Chen XG , et al. Human marrow stromal cell therapy for stroke in rat: neurotrophins and functional recovery. Neurology. 2002;59(4):514‐523. 12196642 10.1212/wnl.59.4.514

[80] Zhang J , Li Y , Chen J , et al. Expression of insulin‐like growth factor 1 and receptor in ischemic rats treated with human marrow stromal cells. Brain Res. 2004;1030(1):19‐27. 15567334 10.1016/j.brainres.2004.09.061

[81] Uccelli A , Benvenuto F , Laroni A , Giunti D . Neuroprotective features of mesenchymal stem cells. Best Pract Res Clin Haematol. 2011;24(1):59‐64. 21396593 10.1016/j.beha.2011.01.004

[82] Voulgari‐Kokota A , Fairless R , Karamita M , et al. Mesenchymal stem cells protect CNS neurons against glutamate excitotoxicity by inhibiting glutamate receptor expression and function. Exp Neurol. 2012;236(1):161‐170. 22561409 10.1016/j.expneurol.2012.04.011

[83] Neuhuber B , Timothy Himes B , Shumsky JS , Gallo G , Fischer I . Axon growth and recovery of function supported by human bone marrow stromal cells in the injured spinal cord exhibit donor variations. Brain Res. 2005;1035(1):73‐85. 15713279 10.1016/j.brainres.2004.11.055

[84] Silva NA , Gimble JM , Sousa N , Reis RL , Salgado AJ . Combining adult stem cells and olfactory ensheathing cells: the secretome effect. Stem Cells Dev. 2013;22(8):1232‐1240. 23316915 10.1089/scd.2012.0524 PMC3614017

[85] Thakor DK , Wang L , Benedict D , Kabatas S , Zafonte RD , Teng YD . Establishing an organotypic system for investigating multimodal neural repair effects of human mesenchymal stromal stem cells. Curr Protoc Stem Cell Biol. 2018;47(1):e58. 30021049 10.1002/cpsc.58

[86] Lu P , Yang H , Jones LL , Filbin MT , Tuszynski MH . Combinatorial therapy with neurotrophins and cAMP promotes axonal regeneration beyond sites of spinal cord injury. J Neurosci. 2004;24(28):6402‐6409. 15254096 10.1523/JNEUROSCI.1492-04.2004 PMC6729552

[87] Zeng X , Zeng YS , Ma YH , et al. Bone marrow mesenchymal stem cells in a three‐dimensional gelatin sponge scaffold attenuate inflammation, promote angiogenesis, and reduce cavity formation in experimental spinal cord injury. Cell Transplant. 2011;20(11–12):1881‐1899. 21396163 10.3727/096368911X566181

[88] Chang C‐P , Chio C‐C , Cheong C‐U , Chao CM , Cheng BC , Lin MT . Hypoxic preconditioning enhances the therapeutic potential of the secretome from cultured human mesenchymal stem cells in experimental traumatic brain injury. Clin Sci (Lond). 2013;124(3):165‐176. 22876972 10.1042/CS20120226

[89] Noronha Nc NDC , Mizukami A , Caliári‐Oliveira C , et al. Priming approaches to improve the efficacy of mesenchymal stromal cell‐based therapies. Stem Cell Res Ther. 2019;10(1):1‐21. 31046833 10.1186/s13287-019-1224-y PMC6498654

[90] Choong C‐J , Baba K , Mochizuki H . Gene therapy for neurological disorders. Expert Opin Biol Ther. 2016;16(2):143‐159. 26642082 10.1517/14712598.2016.1114096

[91] Glavaski‐Joksimovic A , Virag T , T a M , et al. Glial cell line‐derived neurotrophic factor‐secreting genetically modified human bone marrow‐derived mesenchymal stem cells promote recovery in a rat model of Parkinson's disease. J Neurosci Res. 2010;88:2669‐2681. 20544825 10.1002/jnr.22435

[92] Van DJM , Smit‐Oistad IM , Macrander C , et al. Macrophage‐mediated inflammation and glial response in the skeletal muscle of a rat model of familial amyotrophic lateral sclerosis (ALS). Exp Neurol. 2016;277:275‐282. 26775178 10.1016/j.expneurol.2016.01.008 PMC4762214

[93] Xiong N , Zhang Z , Huang J , et al. VEGF‐expressing human umbilical cord mesenchymal stem cells, an improved therapy strategy for Parkinsons disease. Gene Ther. 2011;18(4):394‐402. 21107440 10.1038/gt.2010.152

[94] Krakora D , Mulcrone P , Meyer M , et al. Synergistic effects of GDNF and VEGF on lifespan and disease progression in a familial ALS rat model. Mol Ther. 2013;21(8):1602‐1610. 23712039 10.1038/mt.2013.108 PMC3734670

[95] Pollock K , Dahlenburg H , Nelson H , et al. Human mesenchymal stem cells genetically engineered to overexpress brain‐derived neurotrophic factor improve outcomes in huntington's disease mouse models. Mol Ther. 2016;24(5):965‐977. 26765769 10.1038/mt.2016.12 PMC4881765

[96] Sasaki M , Radtke C , Tan AM , et al. BDNF‐hypersecreting human mesenchymal stem cells promote functional recovery, axonal sprouting, and protection of corticospinal neurons after spinal cord injury. J Neurosci. 2009;29(47):14932‐14941. 19940189 10.1523/JNEUROSCI.2769-09.2009 PMC2825276

[97] Jeong CH , Kim SM , Lim JY , et al. Mesenchymal stem cells expressing brain‐derived neurotrophic factor enhance endogenous neurogensis in an ischemic stroke model. Biomed Res Int. 2014;2014:1‐10. 10.1155/2014/129145 PMC3933216 24672780

[98] Mei J , Niu C . Effects of engineered conserved dopamine neurotrophic factor‐expressing bone marrow stromal cells on dopaminergic neurons following 6‐OHDA administrations. Mol Med Rep. 2015;11(2):1207‐1213. 25373844 10.3892/mmr.2014.2878

[99] Liu H . Neuroprotection by PlGF gene‐modified human mesenchymal stem cells after cerebral ischaemia. Brain. 2006;129(10):2734‐2745. 16901914 10.1093/brain/awl207 PMC2605397

[100] Li T , Ren G , Kaplan DL , Boison D . Human mesenchymal stem cell grafts engineered to release adenosine reduce chronic seizures in a mouse model of CA3‐selective epileptogenesis. Epilepsy Res. 2009;84(2–3):238‐241. 19217263 10.1016/j.eplepsyres.2009.01.002 PMC2746090

[101] Ribeiro TB , Duarte ASS , Longhini ALF , et al. Neuroprotection and immunomodulation by xenografted human mesenchymal stem cells following spinal cord ventral root avulsion. Sci Rep. 2015;5(1):16167. 26548646 10.1038/srep16167 PMC4637826

[102] Lim PK , Patel SA , Gregory LA , Rameshwar P . Neurogenesis: role for microRNAs and mesenchymal stem cells in pathological states. Curr Med Chem. 2010;17(20):2159‐2167. 20423304 10.2174/092986710791299894

[103] Martinez B , Peplow PV . Immunomodulators and microRNAs as neurorestorative therapy for ischemic stroke. Neural Regen Res. 2017;12(6):865‐874. 28761412 10.4103/1673-5374.208540 PMC5514854

[104] Wei GJ , An G , Shi ZW , et al. Suppression of MicroRNA‐383 enhances therapeutic potential of human bone‐marrow‐derived mesenchymal stem cells in treating spinal cord injury via GDNF. Cell Physiol Biochem. 2017;41(4):1435‐1444. 28365701 10.1159/000468057

[105] Tate CC , Chou VP , Campos C , et al. Mesenchymal stromal SB623 cell implantation mitigates nigrostriatal dopaminergic damage in a mouse model of Parkinson's disease. J Tissue Eng Regen Med. 2017;11(6):1835‐1843. 26440859 10.1002/term.2081

[106] Tajiri N , Duncan K , Antoine A , et al. Stem cell‐paved biobridge facilitates neural repair in traumatic brain injury. Front Syst Neurosci. 2014;8:116. 25009475 10.3389/fnsys.2014.00116 PMC4068001

[107] Aizman I , Tirumalashetty BJ , McGrogan M , et al. Comparison of the neuropoietic activity of gene‐modified versus parental mesenchymal stromal cells and the identification of soluble and extracellular matrix‐related neuropoietic mediators. Stem Cell Res Ther. 2014;5(1):29. 24572070 10.1186/scrt418 PMC4055059

[108] Dao MA , Tate CC , Aizman I , McGrogan M , Case CC . Comparing the immunosuppressive potency of naïve marrow stromal cells and Notch‐transfected marrow stromal cells. J Neuroinflammation. 2011;8(1):133. 21982515 10.1186/1742-2094-8-133 PMC3228829

[109] Sullivan R , Duncan K , Dailey T , Kaneko Y , Tajiri N , Borlongan CV . A possible new focus for stroke treatment – migrating stem cells. Expert Opin Biol Ther. 2015;15(7):949‐958. 25943632 10.1517/14712598.2015.1043264 PMC4465850

[110] Steinberg GK , Kondziolka D , Wechsler LR , et al. Clinical outcomes of transplanted modified bone marrow–derived mesenchymal stem cells in stroke. Stroke. 2016;47(7):1817‐1824. 27256670 10.1161/STROKEAHA.116.012995 PMC5828512

[111] Nandy SB , Mohanty S , Singh M , Behari M , Airan B . Fibroblast growth factor‐2 alone as an efficient inducer for differentiation of human bone marrow mesenchymal stem cells into dopaminergic neurons. J Biomed Sci. 2014;21(1):83. 25248378 10.1186/s12929-014-0083-1 PMC4190371

[112] Abdullah RH , Yaseen NY , Salih SM , et al. Induction of mice adult bone marrow mesenchymal stem cells into functional motor neuron‐like cells. J Chem Neuroanat. 2016;77:129–142. 27417692 10.1016/j.jchemneu.2016.07.003

[113] Liang J , Wu S , Zhao H , et al. Human umbilical cord mesenchymal stem cells derived from Wharton's jelly differentiate into cholinergic‐like neurons in vitro. Neurosci Lett. 2013;532(1):59‐63. 23178189 10.1016/j.neulet.2012.11.014

[114] Yu JH , Kim M , Lee M , et al. GABAergic neuronal differentiation induced by brain‐derived neurotrophic factor in human mesenchymal stem cells. Anim Cells Syst (Seoul). 2014;18(1):17‐24.

[115] Salehinejad P , Alitheen NB , Ali AM , et al. Neural differentiation of human umbilical cord matrix‐derived mesenchymal cells under special culture conditions. Cytotechnology. 2015;67(3):449‐460. 25344875 10.1007/s10616-014-9703-6 PMC4371559

[116] Levy YS , Bahat‐Stroomza M , Barzilay R , et al. Regenerative effect of neural‐induced human mesenchymal stromal cells in rat models of Parkinson's disease. Cytotherapy. 2008;10(4):340‐352. 18574767 10.1080/14653240802021330

[117] Somoza R , Juri C , Baes M , Wyneken U , Rubio FJ . Intranigral transplantation of epigenetically induced BDNF‐secreting human mesenchymal stem cells: implications for cell‐based therapies in Parkinson's disease. Biol Blood Marrow Transplant. 2010;16(11):1530‐1540. 20542127 10.1016/j.bbmt.2010.06.006

[118] Yan M , Sun M , Zhou Y , et al. Conversion of human umbilical cord mesenchymal stem cells in Wharton's jelly to dopamine neurons mediated by the Lmx1a and neurturin in vitro: potential therapeutic application for Parkinson's disease in a rhesus monkey model. PLoS One. 2013;8(5):e64000. 10.1371/journal.pone.0064000 PMC3665802 23724014

[119] Choi C‐I , Lee Y‐D , Kim H , et al. Neural induction with neurogenin 1 enhances the therapeutic potential of mesenchymal stem cells in an ALS mouse model. Cell Transplant. 2012;22:1‐41. 22472631 10.3727/096368912X637019

[120] Kim S‐S , Yoo S‐W , Park T‐S , et al. Neural induction with neurogenin1 increases the therapeutic effects of mesenchymal stem cells in the ischemic brain. Stem Cells. 2008;26(9):2217‐2228. 18617687 10.1634/stemcells.2008-0108

[121] Abouelfetouh A , Kondoh T , Ehara K , Kohmura E . Morphological differentiation of bone marrow stromal cells into neuron‐like cells after co‐culture with hippocampal slice. Brain Res. 2004;1029(1):114‐119. 15533322 10.1016/j.brainres.2004.07.092

[122] Lévêque X , Mathieux E , Nerrière‐Daguin V , et al. Local control of the host immune response performed with mesenchymal stem cells: perspectives for functional intracerebral xenotransplantation. J Cell Mol Med. 2015;19(1):124‐134. 25310920 10.1111/jcmm.12414 PMC4288356

[123] Zhang ZX , Guan LX , Zhang K , Zhang Q , Dai LJ . A combined procedure to deliver autologous mesenchymal stromal cells to patients with traumatic brain injury. Cytotherapy. 2008;10(2):134‐139. 18368592 10.1080/14653240701883061

[124] Kishk NA , Gabr H , Hamdy S , et al. Case control series of intrathecal autologous bone marrow mesenchymal stem cell therapy for chronic spinal cord injury. Neurorehabil Neural Repair. 2010;24(8):702‐708. 20660620 10.1177/1545968310369801

[125] Ra JC , Shin IS , Kim SH , et al. Safety of intravenous infusion of human adipose tissue‐derived mesenchymal stem cells in animals and humans. Stem Cells Dev. 2011;20(8):1297‐1308. 21303266 10.1089/scd.2010.0466

[126] Park JH , Kim DY , Sung IY , et al. Long‐term results of spinal cord injury therapy using mesenchymal stem cells derived from bone marrow in humans. Neurosurgery. 2012;70(5):1238‐1247. 22127044 10.1227/NEU.0b013e31824387f9

[127] Bhasin A , Srivastava MVP , Kumaran SS , et al. Autologous mesenchymal stem cells in chronic stroke. Cerebrovasc Dis Extra. 2011;1(1):93‐104. 22566987 10.1159/000333381 PMC3343764

[128] Jarocha D , Milczarek O , Wedrychowicz A , Kwiatkowski S , Majka M . Continuous improvement after multiple mesenchymal stem cell transplantations in a patient with complete spinal cord injury. Cell Transplant. 2015;24(4):661‐672. 25807231 10.3727/096368915X687796

[129] Llufriu S , Sepúlveda M , Blanco Y , et al. Randomized placebo‐controlled phase II trial of autologous mesenchymal stem cells in multiple sclerosis. PLoS One. 2014;9(12):1‐15. 10.1371/journal.pone.0113936 PMC4250058 25436769

[130] Connick P , Kolappan M , Crawley C , et al. Autologous mesenchymal stem cells for the treatment of secondary progressive multiple sclerosis: an open‐label phase 2a proof‐of‐concept study. Lancet Neurol. 2012;11(2):150‐156. 22236384 10.1016/S1474-4422(11)70305-2 PMC3279697

[131] Bonab MM , Sahraian MA , Aghsaie A , et al. Autologous mesenchymal stem cell therapy in progressive multiple sclerosis: an open label study. Curr Stem Cell Res Ther. 2012;7(6):407‐414. 23061813 10.2174/157488812804484648

[132] Milczarek O , Jarocha D , Starowicz‐Filip A , et al. Multiple autologous bone marrow‐derived CD271 + mesenchymal stem cell transplantation overcomes drug‐resistant epilepsy in children. Stem Cells Translational Med. 2018;7:20‐33. 10.1002/sctm.17-0041 PMC5746144 29224250

[133] Bang OY , Lee JS , Lee PH , Lee G . Autologous mesenchymal stem cell transplantation in stroke patients. Ann Neurol. 2005;57(6):874‐882. 15929052 10.1002/ana.20501

[134] Lee JS , Hong JM , Moon GJ , et al. A long‐term follow‐up study of intravenous autologous mesenchymal stem cell transplantation in patients with ischemic stroke. Stem Cells. 2010;28(6):1099‐1106. 20506226 10.1002/stem.430

[135] Honmou O , Houkin K , Matsunaga T , et al. Intravenous administration of auto serum‐expanded autologous mesenchymal stem cells in stroke. Brain. 2011;134(6):1790‐1807. 21493695 10.1093/brain/awr063 PMC3102237

[136] Chen G , Wang Y , Xu Z , et al. Neural stem cell‐like cells derived from autologous bone mesenchymal stem cells for the treatment of patients with cerebral palsy. J Transl Med. 2013;11(1):21. 23351389 10.1186/1479-5876-11-21 PMC3563497

[137] Venkataramana NK , Kumar SKV , Balaraju S , et al. Open‐labeled study of unilateral autologous bone‐marrow‐derived mesenchymal stem cell transplantation in Parkinson's disease. Transl Res. 2010;155(2):62‐70. 20129486 10.1016/j.trsl.2009.07.006

[138] Pal R , Venkataramana NK , Bansal A , et al. Ex vivo‐expanded autologous bone marrow‐derived mesenchymal stromal cells in human spinal cord injury/paraplegia: a pilot clinical study. Cytotherapy. 2009;11(7):897‐911. 19903102 10.3109/14653240903253857

[139] Saito F , Nakatani T , Iwase M , et al. Administration of cultured autologous bone marrow stromal cells into cerebrospinal fluid in spinal injury patients: a pilot study. Restor Neurol Neurosci. 2012;30(2):127‐136. 22232031 10.3233/RNN-2011-0629

[140] Bhanot Y , Rao S , Ghosh D , Balaraju S , C. R. R , K. V. SK . Autologous mesenchymal stem cells in chronic spinal cord injury. Br J Neurosurg. 2011;25(4):516‐522. 21749185 10.3109/02688697.2010.550658

[141] Hlebokazov F , Dakukina T , Ihnatsenko S , et al. Treatment of refractory epilepsy patients with autologous mesenchymal stem cells reduces seizure frequency: an open label study. Adv Med Sci. 2017;62(2):273‐279. 28500900 10.1016/j.advms.2016.12.004

[142] Karamouzian S , Nematollahi‐Mahani SN , Nakhaee N , Eskandary H . Clinical safety and primary efficacy of bone marrow mesenchymal cell transplantation in subacute spinal cord injured patients. Clin Neurol Neurosurg. 2012;114(7):935‐939. 22464434 10.1016/j.clineuro.2012.02.003

[143] Petrou P , Gothelf Y , Argov Z , et al. Safety and clinical effects of mesenchymal stem cells secreting neurotrophic factor transplantation in patients with amyotrophic lateral sclerosis. JAMA Neurol. 2016;73(3):337‐344. 26751635 10.1001/jamaneurol.2015.4321

[144] Gothelf Y , Abramov N , Harel A , Offen D . Safety of repeated transplantations of neurotrophic factors‐secreting human mesenchymal stromal stem cells. Clin Transl Med. 2014;3(1):21. 25097724 10.1186/2001-1326-3-21 PMC4108239

[145] Tian C , Wang X , Wang X , et al. Autologous bone marrow mesenchymal stem cell therapy in the subacute stage of traumatic brain injury by lumbar puncture. Exp Clin Transplant. 2013;11(2):176‐181. 22891928 10.6002/ect.2012.0053

[146] Shichinohe H , Kawabori M , Iijima H , et al. Research on advanced intervention using novel bone marrOW stem cell (RAINBOW): a study protocol for a phase I, open‐label, uncontrolled, dose‐response trial of autologous bone marrow stromal cell transplantation in patients with acute ischemic stroke. BMC Neurol. 2017;17(1):179. 28886699 10.1186/s12883-017-0955-6 PMC5591569

[147] Deng L , Peng Q , Wang H , et al. Intrathecal injection of allogenic bone marrow‐derived mesenchymal stromal cells in treatment of patients with severe ischemic stroke: study protocol for a randomized controlled observer‐blinded trial. Transl Stroke Res. 2019;10(2):170‐177. 29796934 10.1007/s12975-018-0634-y

[148] Park HC , Shim YS , Ha Y , et al. Treatment of complete spinal cord injury patients by autologous bone marrow cell transplantation and administration of granulocyte‐macrophage colony stimulating factor. Tissue Eng. 2005;11(5–6):913‐922. 15998231 10.1089/ten.2005.11.913

[149] Tatsumi K , Ohashi K , Matsubara Y , et al. Tissue factor triggers procoagulation in transplanted mesenchymal stem cells leading to thromboembolism. Biochem Biophys Res Commun. 2013;431(2):203‐209. 23313481 10.1016/j.bbrc.2012.12.134

[150] Law S , Derry S , Moore RA . Triptans for acute cluster headache. Cochrane Database Syst Rev. 2013;7(7):CD008042. 10.1002/14651858.CD008042.pub3 PMC6494511 24353996

[151] Snyder BR , Chiu AM , Prockop DJ , Chan AWS . Human multipotent stromal cells (MSCs) increase neurogenesis and decrease atrophy of the striatum in a transgenic mouse model for Huntington's disease. PLoS One. 2010;5(2):e9347. 20179764 10.1371/journal.pone.0009347 PMC2825266

[152] Boshuizen MCS , Steinberg GK . Stem cell‐based immunomodulation after stroke effects on brain repair processes. Stroke. 2018;49(6):1563‐1570. 29724892 10.1161/STROKEAHA.117.020465 PMC6063361

[153] Oh SK , Jeon SR . Current concept of stem cell therapy for spinal cord injury: a review. Korean J Neurotrauma. 2016;12(2):40‐46. 27857906 10.13004/kjnt.2016.12.2.40 PMC5110917

